# Interferons (*IFN-A/-B/-G*) Genetic Variants in Patients with Mixed Connective Tissue Disease (MCTD)

**DOI:** 10.3390/jcm8122046

**Published:** 2019-11-21

**Authors:** Agnieszka Paradowska-Gorycka, Anna Wajda, Barbara Stypinska, Ewa Walczuk, Marcela Walczyk, Anna Felis-Giemza, Aleksandra Poluch-Lewandowska, Marzena Olesińska

**Affiliations:** 1 Department of Molecular Biology, National Institute of Geriatrics, Rheumatology and Rehabilitation, Warsaw 02-637, Poland; annawajda2046@gmail.com (A.W.); barbara.stypinska@wp.pl (B.S.); ewa.walczuk@spartanska.pl (E.W.); 2 Department of Connective Tissue Diseases, National Institute of Geriatrics, Rheumatology and Rehabilitation, Warsaw 02-637, Poland; marcela.walczyk@gmail.com (M.W.); annafelis@wp.pl (A.F.-G.); poluchola@gmail.com (A.P.-L.); marzena.olesinska@vp.pl (M.O.)

**Keywords:** MCTD, genetics, IFN, pathogenesis

## Abstract

Mixed connective tissue disease (MCTD) is a rare complex autoimmune disease in which autoantigens are recognized by endosomal TLRs. Their activation induces a higher secretion of the type I interferons, IFN-γ and the up-regulation of the INF-inducible genes. The present study aimed to investigate whether SNPs that are located in the IFN-A, IFN-B, and IFN-G genes are associated with MCTD. 145 MCTD patients and 281 healthy subjects were examined for IFN-A, IFN-B, and IFN-G genetic variants by TaqMan SNP genotyping assay. ELISA determined IFN-α/-β/-γ serum levels. Among the seven tested SNPs, four polymorphisms: IFN-A rs10757212, IFN-A rs3758236, IFN-G rs2069705, IFN-G rs2069718, as well as INF-G rs1861493A/rs2069705A/rs2069718G haplotype were significantly associated with a predisposition for MCTD. Raynaud’s phenomenon, erosive arthritis, swollen hands and fingers, and sclerodactyly were significantly more frequently observed in MCTD patients with IFN-G rs2069718 G allele than in patients with IFN-G rs2069718 A allele. We also found that anti-U1-A autoantibodies most frequently occurred in MCTD patients with rs2069718 GA genotype, while the IFN-G rs2069705 AG and rs2069718 GA genotypes might be a marker of anti-Ro60 presence in MCTD patients. Our results indicate that IFN-G genetic variants may be potential genetic biomarkers for MCTD susceptibility and severity.

## 1. Introduction

Autoimmune, systemic connective tissue diseases affect a small percentage of the general population. However, those diseases are chronic, they begin at a relatively young age and lead to progressive disability, which is an important social and medical problem. Mixed connective tissue disease (MCTD) is one of the least frequent among these disorders. The prevalence of MCTD is still uncertain, but it has been estimated at 10/100,000 in the Caucasian population. Sharp et al. first described MCTD in 1972 [1] as a new entity distinct from other connective tissue diseases (CTDs) [2]. In MCTD, like other systemic CTDs, complex interactions between disease-related phenomena, including chronic inflammation, dyslipidemia, thrombotic events, proliferative vascular arteriopathy, production of autoreactive autoantibodies, and humoral autoimmune processes, can be observed [3]. The presence of high-titer of anti-U1-snRNP antibodies (autoantibodies against the U1-small nuclear ribonucleoprotein antigen) in serum is an important hallmark of MCTD, which is a part of the diagnostic criteria for MCTD [3,4,5,6]. Moreover, U1-RNP, which is a complex of U1-snRNA (small nuclear RNA) and small nuclear RNP, are the putative target of autoimmunity in the pathogenesis of MCTD. The U1-RNA molecule and the specific protein, U1 small nuclear ribonucleoprotein 70 kDa (snRNP70 or U1-70K), are both involved in the formation of network interactions between immune cells and their receptors, leading to autoimmunity, inflammation, and consequently to tissue damage [7,8,9,10,11,12]. The U1-snRNP immune complex, which contains its own RNA molecule, might induce an immune response through the involvement of specific autoreactive endosomal Toll-like receptors (TLRs) [7]. MCTD-associated autoantigens are recognized by endosomal TLRs, where activation induces a higher secretion of the type I interferons (IFN I), IFN-γ, up-regulation of the IFN-inducible genes (called IFN signature), as well as higher production of the specific autoantibodies [7,10,13,14]. The type I interferons, such as IFN-α and IFN-β, as well as IFN-γ (which is the only one type II class of interferons), are a family of pleiotropic cytokines with a wide spectrum of effects on adaptive and innate immune processes [15,16,17]. They have a key role in the initiation and progression of autoimmune disease by the promotion of the prolonged survival of activated T lymphocytes, differentiation of the T helper 17 cells (Th17), higher expression of the proinflammatory cytokines and chemokines, as well as by the increase the production of B lymphocyte stimulator and stimulation of B cells proliferation [15,18,19]. Although the detailed etiopathogenesis of systemic CTDs is not established, the growing volume of published studies has confirmed that excessive immunological activation with TLRs and IFNs is very important/one of the crucial pathogenic mechanisms of these diseases [20,21,22,23]. Moreover, the studies also highlighted that the overexpression of IFN-inducible genes (IFN signature) is a marker for patients with active and severe CTD [24].

Given the causative role of the type I and II interferons in human autoimmune diseases, including their role in the initiation of the disease processes and in the molecular pathogenesis of connective tissue diseases [14,25,26], we decided to explore whether single nucleotide polymorphisms (SNPs) that are located in the *IFN-A* (encodes IFN-α), *IFN-B* (encodes IFN-β), and *IFN-G* (encodes IFN-γ) genes may also be associated with MCTD while using a case-control study.

## 2. Materials and Methods

### 2.1. Subjects 

A total of 145 MCTD patients and 281 healthy participants were included in the present study. Healthy participants were recruited from the Regional Center for Blood Donation and Blood Treatment in Warsaw, and they did not have a history of autoimmune and/or inflammatory disease at the time of sampling. All of the MCTD patients were recruited at the Department of Connective Tissue Diseases of the National Institute of Geriatrics, Rheumatology, and Rehabilitation. MCTD patients, as well as healthy subjects, were of Caucasian origin. The MCTD patients met Alarcon-Segovia and Villarreal classification criteria, which were chosen due to their very high specificity for MCTD. Patients with MCTD, who met the classification criteria for two connective tissue diseases at the same time, were excluded from the study. A set of objective and subjective methods to assess the damage in MCTD, as well as disease activity, were developed specifically for the purpose of this study since guidelines to evaluate the disease status of MCTD patients have not been yet established. The activity index, developed at our Institute and based on the Systemic Lupus Erythematosus Disease Activity Index (SLEDAI) for SLE activity assessment, was used to evaluate the clinical activity of MCTD. Applied MCTD activity scale includes the assessment of clinical and laboratory symptoms that indicate active disease. The assigned number of points was calculated for each symptom indicating the activity of the disease, which was found in the last 28 days. The activity index (AI) for a given patient was the sum of all points, with a maximum of 52. In the Mixed Connective Tissue Disease–Activity Index (MCTD-AI), the most important organ symptoms (such as lung involvement, pulmonary hypertension, and vasculitis) that are not present in lupus are taken into account (Table S1 in Supplementary Files). The use of the MCTD-AI scale enables the estimated stratification of patients. Similarly, based on the Systemic Lupus International Collaborating Clinics/American College of Rheumatology (SLICC/ACR) damage index (DI), which assesses damage in SLE, we have developed an MCTD damage scale consisting of clinical symptoms caused by a permanent result of the disease, its treatment or related diseases—Mixed Connective Tissue Disease—Damage Index (MCTD-DI) (Table S2 in Supplementary Files). For every symptom of damage that has occurred during MCTD and has lasted for at least six months, one point was scored, if the episode recurred—two points. The damage index for a given MCTD patient was the sum of all points. SLICC/ACR scale. The identification of antinuclear antibodies (ANA) was performed by indirect immunofluorescence (IF) on the Hep2 cell lines (Euroimmun Polska, Wroclaw, Poland). Autoantibodies to Sm, Ro, La, RibP, PCNA, CENPB, Scl-70, Jo-1, His, dsDNA were measured in sera while using DOT-blot tests (recomLine ANA/ENA, Mikrogen Diagnostik, Neuried, Germany). The presence of anti-U1-RNP was determined by electrochemiluminescence (ECLIA) using streptavidin-coated paramagnetic beads (UNICAP100, Phadia, Sweden). The Research Ethics Committee of the National Institute of Geriatrics, Rheumatology, and Rehabilitation in Warsaw approved the presented clinical investigation (of 14 January 2016). All participants, patients, and healthy donors gave informed written consent and research was conducted in accordance with the Declaration of Helsinki. 

### 2.2. Genomic DNA Extraction

Genomic DNA was extracted from 200 µL of whole blood samples from MCTD patients and healthy subjects while using the QIAamp DNA Blood Mini Kit (Qiagen, Hilden, Germany) in accordance with the manufacturer’s instructions. All of the extracted DNA samples were standardized to give a final concentration of 1.8 ng/uL and stored at −80 °C until the next analyses. 

### 2.3. SNP Selection and Genotyping 

The IFNs polymorphisms were selected based on SNPs position in the gene, possible functional effect, or association with other autoimmune diseases. The SNPs with a minor allele frequency (MAF) <0.05 (<5%) were excluded. The DNA samples were genotyped for SNPs of *IFN-A* rs10757212 and rs3758236, *IFN-B* rs7873167, and rs10964831, and *IFN-G* rs1861493, rs2069705, and rs2069718 using TaqMan allelic discrimination assay (used assay ID: C__11843895_10, C__27475238_10, C__29146577_10, C__31290012_10, C___2683476_10, C__15944115_20, C__15799728_10, respectively, Applied Biosystems, Foster City, CA, USA). The TaqMan SNP genotyping assays were performed by a QuantStudio 5 detection system (Life Technologies, Carlsbad, CA, USA), which were carried out in 384-well plates in a 7.5 μL reaction volume containing 1.8 ng genomic DNA, 0.35 μL assay mix (20×) and 3.75 μL Taqman universal PCR master mix (2×). The reaction was performed with the following amplification protocol: denaturation at 95 °C for 10 min., followed by 40 cycles of denaturation at 92 °C for 15 s, and annealing and extension at 60 °C for 1 min. 

### 2.4. Quantitative Analysis of IFNs

The concentrations of IFN-α/-β/-γ in serum of MCTD and healthy subjects were measured by colorimetric sandwich enzyme-linked immunosorbent assay (ELISA) (SunRed Biological Technology, Shanghai, China). The ELISA test was performed in accordance with the manufacturers’ instructions. For analysis we used 40 µL of serum. The ELISA standard ranges were 6–2000 pg/mL for IFN-α, 8–2000 ng/L for IFN-β, and 2–600 ng/L for IFN-γ. 

### 2.5. Statistical Analysis

All data were presented as a median (interquartile range, IQR) for non-normally distributed continuous variables or as mean ± SD for normally distributed continuous variables. The accordance of genotype distribution with Hardy–Weinberg Equilibrium (HWE) was performed while using the HWE exact test with “genetics” R package (Gregory Warnes, with contributions from Gregor Gorjanc, Friedrich Leisch and Michael Man. (2019); genetics: Population Genetics. R package version 1.3.8.1.1. https://CRAN.R-project.org/package). Logistic regression was used to evaluate genotype and allele distribution between groups (OR, 95% confidence intervals, p-value); the results were adjusted by age and gender accordingly. The analysis was calculated under dominant, codominant, overdominant and recessive, and allelic models. The presence of linkage disequilibrium (LD), as well as a coefficient (D′ and r2) for haplotypes, was carried out while using the SHEsis software, available at http://analysis.bio-x.cn. Differences between tested SNPs, disease activity parameters and cytokines serum concentrations were analyzed while using the Kruskal–Wallis Test, Mann–Whitney test, or analysis of variance for continuous variables and χ^2^ or Fisher exact test for categorical variables. A *p*-value < 0.05 indicated a statistically significant result.

## 3. Results

### 3.1. MCTD Patients Clinical Characteristics and Autoantibodies Profile

A set of objective and subjective methods to assess the damage in MCTD as well as disease activity were developed specifically for the purpose of this study since guidelines to evaluate the disease status of MCTD patients have not been yet established. 

The median value of MCTD-AI was 9.4 points (lowest—0, highest—32 points). In 40% of our MCTD patients, MCTD-DI was at or below six points and these patients had symptoms of organ involvement.

Median value of MCTD -DI was 5 (lowest value—0, highest—12). Damage most commonly affected skin, musculoskeletal and cardiovascular systems, and lungs. Table 1 summarizes the clinical characteristics of MCTD patients. The clinical picture of MCTD was variable. Some symptoms, such as skin lesions typical for SLE, swelling of the hands, arthropathy, inflammation, and involvement of the muscle, were mostly occurring early in the course of the disease. The major clinical features of MCTD patients were Raynaud’s phenomenon (96.7%), hands edema (91.9%), polyarthritis (93.94%), leuco/thrombocytopenia (64.65%), and myopathy (66.67%). 97% of our MCTD patients presented with Raynaud’s phenomenon right at the onset of the disease. ANA in titer >1:320 was detected in 88% of MCTD patients. All of the MCTD patients had anti-U1-RNP antibodies: anti-70K was detected in 76% of patients, anti-A in 84% of patients, and anti-C in 79% of patients (Table 2).

### 3.2. Association of IFN-A, IFN-B and IFN-G Genetic Variants with Risk of MCTD 

In the first step, we determined whether *IFN-A*, *IFN-B*, and *IFN-G* gene variants were associated with MCTD severity. 

Among seven examined SNPs, the distributions of six SNPs genotypes (rs10757212, rs3758236, rs7873167, rs10964831, rs1861493, and rs2069705) were consistent with the Hardy–Weinberg equilibrium (HWE) in both MCTD patients and healthy subjects (Table S3 in Supplementary Files). The *IFN-G* rs2069718 genotype distribution was in HWE in controls, but it showed deviation from HWE in MCTD patients (*p* = 0.02, Table S3 in Supplementary Files). However, the genotyping was randomly repeated on 20% selected samples (10% for MCTD group and 10% for control groups), giving complete conformity of the result. 

Next, we compared the minor allele frequencies (MAF) between our study groups and another European ancestry (EUR) while using 1000 genome bases. The MAF was found to be the lowest for *IFNA* rs3758236 A allele in MCTD patients (A: 0.11) and for *IFN-B* rs7873167 G allele in MCTD patients and controls (G: 0.11); while the highest was for *IFN-G* rs2069705 G allele in the controls (G: 0.52) (Table S3 in Supplementary Files). Generally, the MAF of all examined genetic variants in our cohorts were similar to those in the European ancestry.

In Table 3 we have shown genotype distribution and allele frequencies for seven genotyped SNPs in MCTD patients and healthy subjects. Four different genetic models: codominant, dominant, recessive, and overdominant, were tested to explore the relation of INFs genetic variants with MCTD risk. Among the seven tested SNPs, four polymorphisms (*IFN-A* rs10757212, *IFN-A* rs3758236, *IFN-G* rs2069705, *IFN-G* rs2069718) were significantly associated with a predisposition for MCTD. For *IFN-A* rs10757212 and *IFN-G* rs2069705, the recessive model revealed significant differences between the MCTD patients and healthy subjects. We observed that the *IFN-A* rs10757212 GG + GA genotype and *IFN-G* rs2069705 AA + AG genotype was significantly higher in MCTD patients when compared to controls (*p* = 0.01 and *p* = 0.03, respectively). For *IFN-A* rs3758236 the codominant, dominant, and recessive models yielded significant differences between MCTD patients and controls. The *IFN-A* rs3758236 TT genotype under the codominant and dominant models and *IFN-A* rs3758236 TT + TA genotype under the recessive model were more frequently observed in MCTD patients than in the controls (*p* = 0.03, *p* = 0.04 and *p* = 0.02, respectively). The *IFN-A* rs3758236 A allele were also more frequently observed in MCTD patients than in healthy subjects (89% vs 82%, *p* = 0.04). For *IFN-G* rs2069718 GG genotype under the codominant model and rs2069718 GG + GA genotype under the recessive models have shown an association with MCTD risk (*p* = 0.02 and *p* = 0.008, respectively). The frequencies of the G allele of the *IFN-G* rs2069718 also significantly differed between MCTD patients and healthy subjects (60% vs 48%, *p* = 0.008). With respect to *IFN-B* rs7873167, IFN-B rs10964831, and *IFN-G* rs1861493, there were no differences in the genotype distribution and allele frequencies between MCTD patients and healthy subjects (*p* > 0.05, Table 3).

### 3.3. Genetic Association of IFNs Haplotypes with MCTD

The SHEsis program has been used. The analysis revealed a quite strong LD between *IFN-G* rs2069705 and rs2067918 with D’ = 0.77 and *r*^2^ = 0.5. In other examined genetic variants, we observed a weak LD, as the *r*^2^ value between all of them is considerably low (*r*^2^ < 0.3).

The following sequence: *IFN-A* rs10757212 G/A, *IFN-A* rs3758236 T/A, *IFN-B* rs7873167 T/G, IFN-B rs10964831 A/T were used to create the haplotypes because the *INF-A* and *INF-B* genes are located on the same chromosome 9. Sixteen potential haplotypes were formed in both MCTD patients and healthy subjects; however, the haplotypes with frequency of <0.03 are ignored (Table 4). The most frequent haplotype observed in both studied groups was GTTA (59% of MCTD patients and 50% of HC, *p* = 0.07). Other haplotypes occur with moderate frequency. The frequency of ATTA and GATA haplotypes was significantly higher in healthy subjects than in MCTD patients (*p* = 0.03 and *p* = 0.01, respectively), thus conferring disease protection nature to these haplotypes. 

For *INF-G*, gene haplotypes were separately created because this gene is located on chromosome 12. To create the haplotypes, *INF-G* polymorphisms were tested in the following sequence: rs1861493 A/G, rs2069705 A/G, and rs2069718 G/A. We observed that eight potential haplotypes were created; however, the haplotypes with frequency of <0.03 were ignored (Table 4). The most common haplotype was AAG, which was observed in 58% of MCTD patients and in 36% of healthy subjects. This AAG haplotype was also significantly associated with the risk for MCTD (*p* < 0.0001). In contrast, the AAA and AGG haplotypes, which were the minor haplotypes in MCTD patients, were significantly associated with a lower risk of MCTD (AAA: 3% in MCTD vs 13% in HC, *p* = 0.0003; AGG: 0.4% in MCTD vs 10% in HC, *p* < 0.0001; Table 4). 

### 3.4. Correlation of the IFNs Genetic Variants with Clinical Variables in Patients with MCTD

Hence, it could conceivably be hypothesized that all three interferons, *IFN-A*, *IFN-B,* and *IFN-G*, may be good candidate genes to play a part in MCTD autoimmune process, we analyzed whether examined IFNs genetic variants may have an impact on MCTD phenotype. We tested the genotype frequencies of IFNs polymorphisms, in different genetic models (codominant (Table 5), dominant (Table 6) and recessive (Table 7)). Table 5, Table 6 and Table 7 only present significant association or association with tendency. Pulmonary fibrosis and poikiloderma were more frequently observed in MCTD patients with *IFN-A* rs3758236 TT genotype than in MCTD patients with *IFN-A* rs3758236 TA or AA genotype (Table 5, *p* = 0.03 and *p* = 0.04, respectively). Rheumatoid factor was more frequent in MCTD patients with *IFN-B* rs7873167 TT genotype when comparing to MCTC patients with other genotypes (*p* = 0.01). The MCTD patients with *IFN-B* rs10964831 TT genotype were characterized by significantly higher damage index than other MCTD patients (TT: 33.5 ± 31.5, AT: 12.577 ± 3.145, AA: 7.879 ± 1.364, *p* = 0.01). In MCTD patients with *IFN-G* rs1861493 AG genotype, we observed more frequent occurrence of rheumatoid factors (Table 5, *p* = 0.03) as well as a tendency towards the more frequent occurrence of lymphadenopathy (Table 5, *p* = 0.06) and enlargement of lymph nodes (Table 5, *p* = 0.06) when compared to MCTD patients with *IFN-G* rs1861493 AA or GG genotypes (Table 5). Moreover, in the carriers of the polymorphic *IFN-G* rs1861493 G allele Sjogren’s syndrome and poikiloderma were present considerably more frequently than in patients with two wild-type alleles (Table 6, *p* = 0.02 and *p* = 0.03, respectively). The swollen hands and fingers were more frequently observed in MCTD patients with *IFN-G* rs2069705 A allele than in the carriers of *IFN-G* rs2069705 GG genotype (Table 7, *p* = 0.03). In the carriers of *IFN-G* rs2069705 A allele, we also observed a tendency to the more frequent presence of sclerodactyly (Table 7, *p* = 0.07). In contrast, in the carriers of *IFN-G* rs2069705 G allele, we observed a tendency for the presence of pericarditis and poikiloderma to appear more frequently (Table 6, *p* = 0.07 and *p* = 0.06, respectively). Additionally, Raynaud’s Phenomenon and erosive arthritis were more often present in the MCTD patients with *IFN-G* rs2069718 GA genotype than in the MCTD patients with other genotypes. Moreover, the erosive arthritis was not present in MCTD patients with *IFN-G* rs2069718 AA genotype (Table 5). Furthermore, swollen hands and fingers, as well as sclerodactyly, were more frequently observed in MCTD patients with *IFN-G* rs2069718 G allele than in MCTD patients with IFN-G rs2069718 A allele (Table 7, both *p* = 0.04). 

### 3.5. INFs Gene Polymorphisms Association with Serologic Profile of MCTD Patients

Analysis of the association between the *IFN-G* genetic variants and autoantibody profiles in patients with MCTD was the next stage of present research. The *IFN-A* and *IFN-B* gene polymorphisms, which were examined in this study, were excluded for this analysis because we have only 1 or 2 patients with polymorphic genotype. The frequencies of all examined *IFN-G* polymorphisms were compared between the MCTD patients with the presence of autoantibodies and MCTD patients without autoantibodies (Table 8a,b). The results showed an significant association between *IFN-G* rs1861493 A/G and RF (*p* = 0.03) and anti-DNA (*p* = 0.01) presence; between *IFN-G* rs2069705 A/G genetic variant and anti-U1-A (*p* = 0.03), anti-Ro60 (*p* = 0.009), and anti-DNA (*p* = 0.02) presence; as well as between IFN-G rs2069718 G/A and RF (*p* = 0.08), anti-U1-RNP (0.056), anti-Ro60 (*p* = 0.02), and anti-DNA (*p* = 0.04) presence. Anti-U1-A most frequently occurred in MCTD patients with rs2069705AG and rs2069718 GA genotype. In MCTD patients with the *IFN-G* rs1861493 AG genotype, the rheumatoid factor was present more often than in other genotypes (*p* = 0.03). In contrast, in MCTD patients with the *IFN-G* rs1861493 GG genotype, we did not observe anti-Ro60 antibodies. Similarly, in MCTD patients with the *IFN-G* rs2069705AA and *IFN-G* rs2069718 GG genotypes, we did not observe anti-CCP antibodies.

We also observed that *IFN-G* rs1891493 AG might be a marker of anti-CCP presence in MCTD patients; furthermore, the *IFN-G* rs2069705 AG and *IFN-G* rs2069718 GA genotypes might be a marker of anti-Ro60 presence in MCTD patients (Table 8b).

### 3.6. Variation in IFN-α/-β/-γ Serum Levels in Relation to IFN-A/-B/-G Gene Polymorphisms

The last part of our analysis was to determine the impact of IFNs genetic variants on interferons serum levels in patients with MCTD and controls. In our study, we observed that IFN-α showed the highest level in serum in MCTD patients (median: 650.7 pg/mL), while IFN-γ was at the lowest level in serum (median: 31.9 ng/L). Analysis of IFN-α/-β/-γ levels in serum in MCTD patients and healthy subjects in relation to all examined IFN-A/-B/-G genetic variants revealed no association between IFN-A/-B/-G different genotypes and IFN-α/-β/-γ levels in serum among MCTD patients and as well as in the control groups.

## 4. Discussion

The current investigation is the first, which showed the association between type I and II interferons genetic variants and MCTD. The fact that MCTD is a rare complex disease makes the recruitment of large sample size challenging. Therefore, we believe that our group of 145 MCTD patients with a very detailed characteristic is the added value of this work and the findings of this study may also be significant for clinical practice. The interferons play a very important role in the pathogenesis of the autoimmune diseases and they are produced in response to inflammatory stimuli. The interaction between type I (IFN-α and IFN-β) as well as type II (IFN-γ) interferons and immune cells leads to the induction of many aspects of the immune response. Type I and II interferons have both pleiotropic cellular effects, such as cell cycle, proliferation, and differentiation regulation, and antiproliferative effects, such as the regulation of DNA replication and modulation of gene expression [27]. The role of interferons in the braking of the immune system homeostasis and leading to the initiation and progression of the immune processes makes IFNs genes a potential candidate for MCTD susceptibility/severity.

The present study demonstrates, for the first time, the association between *IFN-A, IFN-B,* and *IFN-G* genetic variants and MCTD risk, phenotype, and serological profile. Several studies have reported that the dysregulation of the IFN system occurs in connective tissue diseases, such as systemic lupus erythematosus (SLE), systemic sclerosis (SSc), or rheumatoid arthritis (RA) [24]. Genome wide-association studies (GWAS) identified IFN and IFN-inducible genes as loci that contribute to CTDs susceptibility and/or severity [26,28,29,30,31,32]. To date, there is no GWAS published for MCTD. Our results showed a significant association of the *IFN-A* rs10757212, *IFN-A* rs3758236, *IFN-G* rs2069705, and *IFN-G* rs2069718 gene polymorphisms with the occurrence of MCTD. The present study also demonstrated that *INF-G* rs1861493A/rs2069705A/rs2069718G haplotype, which was observed in 58% of MCTD patients, was significantly associated with MCTD risk. Our results indicated that *IFN-A,* as well as *IFN-G* genetic variants, might be potential genetic biomarkers for MCTD susceptibility. Although we found that the IFN-α/-β/-γ levels in serum in MCTD patients were higher than in healthy subjects in the present study, we did not detect the association between interferons genetic variants and IFN serum levels. This may be due to a small sample size of our study group or that the SNPs located in the IFNs genes may have an impact on the changing structure of their protein rather than the regulation of serum protein levels. Moreover, changes in the protein structure can lead to a modification of the IFN affinity for its receptors that are involved in the immune pathways. On the other hand, epigenome-wide association studies (EWAS) in MCTD patients [33] and Genome-wide transcriptional profiling in SLE or SSc patients [24,34,35] confirmed the overexpression of IFN-inducible genes, known as the “interferon (IFN) signature” in these patients. The overexpression of interferon signature occurs early in the hematopoietic process and correlates with disease activity. Our results also demonstrated that the *IFN-G* genetic variant at position rs2069718 G/A might be associated with some clinical symptoms of MCTD, such as Raynaud’s Phenomenon, erosive arthritis, swollen hands and fingers, and sclerodactyly. The clinical picture of MCTD develops over time. Usually, the first symptom is Raynaud’s Phenomenon, which is accompanied by swollen hands and fingers, erosive arthritis, and skin involvement. Raynaud’s Phenomenon and erosive arthritis, as two of the most devastating complications of MCTD, at the time blood sampling were observed in 97% and 94% of MCTD patients, respectively. In our study, we observed that both Raynaud’s Phenomenon and erosive arthritis were more often present in MCTD patients with the *IFN-G* rs2069718 GA genotype than in MCTD patients with other genotypes. Moreover, the erosive arthritis was not present in MCTD patients with the *IFN-G* rs2069718 AA genotype. Further, swollen hands and fingers, as well as sclerodactyly, were considerably more frequently observed in MCTD patients with *IFN-G* rs2069718 G allele than in MCTD patients with IFN-G rs2069718 A allele. The characteristics of autoimmune CTDs include the presence of autoantibodies, which are involved in the disease pathogenesis, in combination with particular clinical parameters. In MCTD patients, different autoantibodies are described and they may be a partial explanation of diverse clinical phenotype. Autoantibodies against RNA-associated proteins, such as anti-U1-RNP, Anti-Sjögren’s-syndrome-related antigen A (anti-SSA; anti-Ro60; and, anti-Ro52), and anti-Sjögren’s syndrome type B (anti-SSBalso known asanti-La) can enhance the immune response. The most common antibodies that are observed in MCTD patients are anti-U1-RNP (present in all our patients), anti-U1-70K (present in 76% of our patients), anti-U1-A (present in 84% of our patients), and anti-U1-C (present in 79% of our patients). In the anti-RNP response, autoantibodies against U1-70K and Sm-B/B proteins appear first, and then autoantibodies directed against U1-A and -C and at the end autoantibodies directed against Sm-D [9]. In the present study, we observed that the anti-U1-A autoantibodies were the most frequently occurring in MCTD patients with rs2069718 GA genotype. Anti-U1-RNP antibodies through the upregulation of the intracellular adhesion molecule-1, endothelial leukocyte adhesion molecule-1, and major histocompatibility complex (MHC) class II molecules expression may lead to the hyperplasia, obliterated vasculopathy, and pulmonary arterial hypertension (PAH). Moreover, in MCTD patients, autoantibody titers against the U1-RNP may correlate with disease activity [36]. Autoantibodies against RNA binding protein Ro60, which regulate IFN stimulated gene expression [16], were present in 10% of our MCTD patients. Our results indicate that IFN-G rs2069705 AG and rs2069718 GA genotypes may be markers of anti-Ro60 presence in MCTD patients (anti-Ro60 were not present in MCTD patients with other IFN-G rs2069705 and rs2069718 genotypes). 

However, our study has severallimitations. First, our sample size is relatively small and might lead to a limited statistical impact on the accuracy and precision of the results. However, MCTD is a very rare disease and the recruitment of a large sample size is challenged. We determined the alpha coefficient = 0.05 to avoid the type 1 error. This is a value that allows for 95% to avoid the first type of error. Although in some analyses (e.g., autoantibodies profiles) we found statistical significance results, the small number of selected subgroups of MCTD patients does not allow for high test power. The second limitation of this study is the absence of precise measures of clinical activity of MCTD—for the purpose of this study we constructed a set of objective and subjective methods to assess the damage in MCTD as well as disease activity. These methods were developed based on the tools that were employed in SLE. Third, our study was performed in Polish Caucasians subjects only. Future prospective studies involving other populations with different ethnicities are needed and would be interesting. 

## 5. Conclusions

In conclusion, our results add novel evidence in elucidating the genetic predisposition among MCTD patients. This was the first study to show that IFN-A and IFN-G play unique roles in the development of MCTD. What is more, we first demonstrated that IFN-B, as well as IFN-G, play a special/exceptional role in the MCTD phenotype. Our study indicates that IFN-G genetic variants could be potential biomarkers for MCTD susceptibility and severity.

## Figures and Tables

**Table 1:** Clinical and biochemical characteristics of the study group.

Parameter		MCTD Patients	
		*N*	*n* (%)
sex	men	99	15 (15.15%) 84 (84.85%)
	women		
Damage index (median range (min–max)) MCTD-DI		83	5 (0–12)
Activity index (median range (min–max)) MCTD-AI		83	9.4 (0–32)
Disease duration (months; median range (min–max))		99	96.0 (10-420)
course of disease: single-phase		99	12 (12.12%) 28 (28.28%) 59 (59.6%)
course of disease: multiphase			
course of disease: chronic progressive			
Raynaud’s phenomenon		99	96 (96.97%)
Sjogren’s syndrome		99	22 (22.2%)
Hands edema		99	91 (91.92%)
deformative and/or erosive arthritis		99	13 (13.13%)
Poikiloderma		99	30 (30.3%)
Polyarthritis		99	93 (93.94%)
swollen hand and fingers		99	89 (89.8%)
Lymphadenopathy		99	26 (26.26%)
facial erythema		99	42 (42.42%)
pericarditis/pleuritis		99	16 (16.16%)
Leuco/thrombocytopenia		99	64 (64.65%)
Sclerodactyly		99	34 (34.34%)
pulmonary fibrosis		99	30 (30.3%)
Pulmonary arterial hypertension (PAH)		99	12 (12.12)
esophageal involvement/dysphagia		99	22 (22.22%)/21 (21.21%)
Myopathy		99	66 (66.67%)
Cardiomyopathy or ventricular dysfunction documented by echocardiography (chamber?)		99	4 (4.04%)
Therapeutic profiles:			
Azathioprine			6 (6%)
Methotrexate			23 (23%)
Prednisone			76 (77%)
Chloroquine			58 (59%)

*N*—number of patients with clinical information; *n*—number of patients with positive clinical manifestation.

**Table 2:** Autoantibody profile in the mixed connective tissue disease (MCTD) group.

Autoantibody Target	MCTD Patients	
	*N*	*n* (%)
RF	97	36 (37%)
anti-CCP	70	3 (4%)
anti-U1-RNP	99	99 (100%)
anti-70K	99	75 (76%)
anti-A	99	83 (84%)
anti-C	98	78 (79%)
anti-SmB	97	29 (30%)
anti-SmD	99	6 (6%)
anti-Ro60	99	10 (10%)
anti-Ro52	99	24 (24%)
anti-La	99	2 (2%)
anti-RibP	99	3 (3%)
anti-PCNA	99	1 (1%)
anti-CENP B	99	0
anti-Scl-70	99	2 (2%)
anti-Jo-1	99	1 (1%)
anti-His	98	13 (13%)
anti-dsDNA	99	13 (13%)

*N*—number of patients with clinical information; *n*—number of patients with positive clinical manifestation;.RF—rheumatoid factor; anti-CCP—anti-cyclic citrullinated peptide autoantibodies, aCCP; anti-RibP—anti-protein ribosomal P; anti-PCNA—antibodies directed against proliferating cell nuclear antigen; anti-CENP B—anti-Centromere Protein B; anti-Scl-70 - anti-topoisomerase I; anti-Jo-1—antihistidyl transfer RNA [t-RNA] synthetase; anti-dsDNA—anti-double stranded DNA.

**Table 3:** Genotype distribution and allele frequencies of the interferons (IFNs) polymorphisms in MCTD patients and healthy subjects (adjusted by sex and age).

Genetic Model	Genotype	MCTD *n* (%)	Control *n* (%)	OR	*p*-Value
***IFN-A* rs10757212**					
**Codominant**	GG	65 (67%)	173 (64%)	1	**0.05**
	GA	31 (32%)	79 (29%)	1.02 (0.6–1.75)	
	AA	1 (1%)	19 (7%)	0.15 (0.02–1.15)	
**Dominant**	GG	65 (67%)	173 (63.8%)	1	0.5
	GA + AA	32 (33%)	98 (36.2%)	0.86 (0.51–1.45)	
**Recessive**	GG + GA	96 (99%)	252 (93%)	1	**0.01**
	AA	1 (1%)	19 (7%)	0.14 (0.02–1.13)	
**Overdominant**	GG + AA	66 (68%)	192 (71%)	1	0.6
	GA	31 (32%)	79 (29%)	1.11 (0.66–1.9)	
**Allele**	G	161 (83%)	425 (78%)	1	
	A	33 (17%)	117 (22%)	0.74 (0.46–1.14)	0.2
***IFN-A* rs3758236**					
**Codominant**	TT	74 (79%)	192 (68%)	1	**0.03**
	TA	19 (20%)	78 (28%)	0.66 (0.36–1.2)	
	AA	1 (1%)	11(4%)	0.14 (0.02–1.12)	
**Dominant**	TT	74 (79%)	192 (68%)	1	**0.04**
	TA + AA	20 (21%)	89 (32%)	0.56 (0.31–1.01)	
**Recessive**	TT + TA	93 (99%)	270 (96%)	1	**0.02**
	AA	1(1%)	11 (4%)	0.15 (0.02–1.24)	
**Overdominant**	TT + AA	75 (80%)	203 (72%)	1	0.2
	TA	19 (20%)	78 (28%)	0.71 (0.39–1.29)	
**Allele**	T	167 (89%)	462 (82%)	1	
	A	21 (11%)	100 (18%)	0.58 (0.35–0.96)	**0.04**
***IFN-B* rs7873167**					
**Codominant**	TT	77 (79%)	221 (80%)	1	0.7
	TG	18 (19%)	53 (19%)	1.06 (0.57–1.98)	
	GG	2 (2%)	3 (1%)	2.18 (0.32–14.86)	
**Dominant**	TT	77 (79%)	221 (80%)	1	0.7
	TG + GG	20 (21%)	56 (20%)	1.12 (0.61–2.05)	
**Recessive**	TT + TG	95 (98)	274 (99%)	1	0.4
	GG	2 (2%)	3 (1%)	2.16 (0.32–14.63)	
**Overdominant**	TT + GG	79 (81%)	224 (81%)	1	0.8
	TG	18 (19%)	53 (19%)	1.05 (0.56–1.95)	
**Allele**	T	172 (89%)	495 (89%)	1	
	G	22 (11%)	59 (11%)	1.07 (0.64–1.80)	0.7
***IFN-B* rs10964831**					
**Codominant**	AA	66 (70%)	163 (73%)	1	0.6
	AT	26 (28%)	59 (26%)	1.15 (0.64–2.05)	
	TT	2 (2%)	3 (1%)	2.45 (0.35–17.21)	
**Dominant**	AA	66 (70%)	163 (73%)	1	0.5
	AT + TT	28 (30%)	62 (27%)	1.2 (0.68–2.12)	
**Recessive**	AA + AT	92 (98%)	222 (99%)	1	0.3
	TT	2 (2%)	3 (1%)	2.39 (0.34–16.66)	
**Overdominant**	AA + TT	68 (72%)	166 (74%)	1	0.6
	AT	26 (28%)	59 (26%)	1.13 (0.63–2.02)	
**Allele**	A	158 (84%)	385 (85%)	1	
	T	30 (16%)	65 (15%)	1.12 (0.70–1.80)	0.6
***IFN-G* rs1861493**					
**Codominant**	AA	38 (48%)	33 (55%)	1	0.5
	AG	34 (42%)	21 (35%)	1.56 (0.69–3.53)	
	GG	8 (10%)	6 (10%)	1.03 (0.27–3.98)	
**Dominant**	AA	38 (48%)	33 (55%)	1	0.3
	AG + GG	42 (52%)	27 (45%)	1.44 (0.66–3.11)	
**Recessive**	AA + AG	72 (90%)	54 (90%)	1	0.8
	GG	8 (10%)	6 (10%)	0.84 (0.23–3.11)	
**Overdominant**	AA + GG	46 (58%)	39 (65%)	1	0.2
	AG	34 (42%)	21 (35%)	1.55 (0.7–3.41)	
**Allele**	A	110 (69%)	87 (73%)	1	
	G	50 (31%)	33 (27%)	1.20 (0.71–2.02)	0.5
***IFN-G*** **rs2069705**					
**Codominant**	AA	36 (38%)	68 (32%)	1	0.09
	AG	49 (52%)	99 (47%)	0.86 (0.49–1.51)	
	GG	10 (10%)	45 (21%)	0.41 (0.18–0.95)	
**Dominant**	AA	36 (38%)	68 (32%)	1	0.2
	AG + GG	59 (62%)	144 (68%)	0.73 (0.42–1.25)	
**Recessive**	AA + AG	85 (90%)	167 (79%)	1	**0.03**
	GG	10 (10%)	45 (21%)	0.45 (0.21–0.98)	
**Overdominant**	AA + GG	46 (48%)	113 (53%)	1	0.6
	AG	49 (52%)	99 (47%)	1.12 (0.67–1.88)	
**Allele**	A	121 (64%)	235 (55%)	1	
	G	69 (36%)	189 (45%)	0.71 (0.50–1.01)	0.06
***IFN-G*** **rs2069718**					
**Codominant**	GG	31 (32%)	35 (23%)	1	**0.02**
	GA	55 (57%)	77 (50%)	0.83 (0.43–1.59)	
	AA	11 (11%)	41 (27%)	0.32 (0.13–0.79)	
**Dominant**	GG	31 (32%)	35 (23%)	1	0.1
	GA + AA	66 (68%)	118 (77%)	0.66 (0.35–1.23)	
**Recessive**	GG + GA	86 (89%)	112 (73%)	1	**0.008**
	AA	11 (11%)	41 (27%)	0.36 (0.16–0.8)	
**Overdominant**	GG + AA	42 (43%)	76 (50%)	1	0.3
	GA	55 (57%)	77 (50%)	1.28 (0.73–2.25)	
**Allele**	G	117 (60%)	147 (48%)	1	
	A	77 (40%)	159 (52%)	0.61 (0.42–0.88)	**0.008**

*p* ≤ 0.05 was considered as significant, all significant *p*-value was bolded.

**Table 4:** Haplotype analysis results among two single nucleotide polymorphisms (SNPs) in IFN-A locus, two SNPs in IFN-B locus and three SNPs in INF-G locus.

**Haplotype** **IFN-A rs10757212 G/A, IFN-A rs3758236 T/A, IFN-B rs7873167 T/G, IFN-B rs10964831 A/T**	**Frequency**	**MCTD Patients**	**Controls**	***p*-Value**
GTTA	0.53	0.59	0.50	0.07
GTTT	0.11	0.13	0.10	0.2
AATA	0.06	0.08	0.05	0.5
GTGA	0.06	0.06	0.06	0.7
ATTA	0.10	0.05	0.12	**0.03**
GATA	0.07	0.02	0.08	**0.01**
**Haplotype** **IFN-G rs1861493 A/G, rs2069705 A/G, rs2069718 G/A**	**Frequency**	**MCTD Patients**	**Controls**	***p*-Value**
AAG	0.46	0.58	0.36	**<0.0001**
GAG	0.04	0.05	0.03	0.1
AGA	0.1	0.1	0.09	0.4
GGA	0.25	0.23	0.25	0.3
AAA	0.08	0.03	0.14	**0.0003**
AGG	0.05	0.004	0.1	**<0.0001**

*p* values in bold face are considered as significant.

**Table 5:** Correlation between *IFN-A, IFN-B, IFN-G* genotypes (codominant model), and clinical pathology parameters in MCTD.

Parameter/Genotype	MCTD *n* (%)	OR, 95%CI	*p*-Value
**IFN-A rs3758236**			
**pulmonary fibrosis**			
TT	24 (88.9%)	1	**0.03**
TA	2 (7.4%)	0.25 (0.05–1.15)	
AA	1 (3.7%)		
**poikilodermy**			
TT	19 (65.5%)	1	**0.04**
TA	10 (34.5%)	3.22 (1.14–9.11)	
AA	0 (0%)	0	
**IFN-B rs7873167**			
**RF presence**			
TT	24 (68.6%)	1	**0.01**
TG	11 (31.4%)	3.97 (1.31–12.01)	
GG	0 (0%)	0	
**lymphadenopathy**			
TT	18 (69.2%)	1	0.06
TG	6 (23.1%)	1.64(0.54–4.99)	
GG	2 (7.7%)		
**IFN-G rs1861493**			
**RF presence**			
AA	9 (31%)	1	**0.03**
AG	18 (62.1%)	3.62 (1.33–9.92)	
GG	2 (6.9%)	1.29(0.21–7.82)	
**Sjogren’s Syndrome**			
AA	3 (20%)	1	0.06
AG	9 (60%)	4.29(0.98–18.72)	
GG	3 (20%)	6.67(0.89–49.67)	
**lymphadenopathy**			
AA	6 (30%)	1	0.06
AG	13 (65%)	3.3(108–10.05)	
GG	1 (5%)	0.76 (0.08–7.37)	
**poikilodermy**			
AA	7 (29.2%)	1	0.09
AG	14 (58.3%)	3.1(1.07–9.01)	
GG	3 (12.5%)	2.66(0.51–13.83)	
**lymphadenopathy**			
AA	6 (30%)	1	0.06
AG	13 (65%)	3.3 (1.08–10.05)	
GG	1 (5%)	0.76 (0.08–7.37)	
**IFN-G rs2069705**			
**swollen hands and finger**			
AA	34 (39.1%)	1	0.09
AG	46 (52.9%)	0.9 (0.14–5.7)	
GG	7 (8%)	0.14 (0.02–0.98)	
**sclerodactyly**			
AA	10 (32.3%)	1	0.09
AG	20 (64.5%)	1.79 (0.71–4.52)	
GG	1 (3.2%)	0.29 (0.03–2.58)	
**IFN-G rs2069718**			
**Raynaud phenomenon**			
GG	31 (33%)	1	**0.03**
GA	54 (57.4%)	0	
AA	9 (9.6%)	0	
**erosive arthritis**			
GG	1 (7.7%)	1	**0.02**
GA	12 (92.3%)	8.37 (1.03–67.86)	
AA	0 (0%)	0	

*p* values in bold face are considered as significant.

**Table 6:** Correlation between *IFN-A, IFN-B, IFN-G* genotypes (dominant model), and clinical pathology parameters in MCTD.

**IFN-A rs3758236**			
**poikilodermy**			
TT	19 (65.6%)	1	**0.04**
TA + AA	10 (34.5%)	2.89 (1.04–8.03)	
**IFN-G rs1861493**			
**Sjogren’s syndrome**			
AA	3 (20%)	1	**0.02**
AG + GG	12 (80%)	4.71 (1.14–19.48)	
**poikilodermy**			
AA	7 (29.2%)	1	**0.03**
AG + GG	17 (70.8%)	3.01 (1.08–8.4)	
**IFN-G rs2069705**			
**pericarditis**			
AA	3 (18.8%)	1	0.07
AG + GG	13 (81.2%)	3.11 (0.82+11.79)	
**poikilodermy**			
AA	7 (24.1%)	1	0.06
AG + GG	22 (75.9%)	2.46 (0.92–6.56)	

*p* values in bold face are considered as significant.

**Table 7:** Correlation between *IFN-A, IFN-B, IFN-G* genotypes (recessive model) and clinical pathology parameters in MCTD.

**IFN-G rs2069705**			
**swollen hands and fingers**			
AA + AG	89 (92%)	1	**0.03**
GG	7 (8%)	0.15 (0.03 + 0.74)	
**Sclerodactyly**			
AA + AG	30 (96.8%)	1	0.08
GG	1 (3.2%)	0.2 (0.02+1.69)	
**IFN-G rs2069718**			
**swollen hands and fingers**			
GG + GA	81 (91%)	1	**0.04**
AA	8 (9%)	0.16 (0.03+0.82)	
**Sclerodactyly**			
GG + GA	31 (96.9%)	1	**0.04**
AA	1 (3.1%)	0.18 90.02+1.45)	

*p* values in bold face are considered as significant.

**Table:** (**a**)

Genotype	Anti-U1-70K		Anti-U1-A		Anti-U1-C		Anti-SmB		Anti-SmD	
	(−)	(+)	(−)	(+)	(−)	(+)	(−)	(+)	(−)	(+)
**INF-G rs1861493 A/G**										
**AA**	12 (32%)	26 (68%)	7 (18%)	31 (82%)	8 (21%)	30 (79%)	27 (71%)	11 (29%)	36 (95%)	2 (5%)
**AG**	6 (18%)	28 (82%)	5 (15%)	29 (85%)	5 (15%)	29 (85%)	21 (62%)	13 (38%)	31 (91%)	3 (9%)
**GG**	1 (13%)	7 (87%)	3 (38%)	5 (62%)	2 (25%)	6 (75%)	6 (75%)	2 (25%)	7 (87%)	1 (13%)
***p*-value**	0.3		0.3		0.7		0.6		0.7	
**INF-G rs2069705 A/G**										
**AA**	9 (25%)	27 (75%)	8 (22%)	28 (78%)	5 (14%)	31 (86%)	23 (64%)	13 (36%)	34 (94%)	2 (6%)
**AG**	13 (27%)	36 (73%)	4 (8%)	45 (92%)	9 (18%)	40 (82%)	33 (67%)	15 (31%)	34 (94%)	2 (6%)
**GG**	3 (30%)	7 (70%)	4 (40%)	6 (60%)	4 (40%)	6 (60%)	8 (80%)	2 (20%)	9 (90%)	1 (10%)
***p*-value**	0.9		**0.03**		0.1		0.6		0.9	
**IFN-G rs2069718 G/A**										
**GG**	8 (26%)	23 (74%)	6 (19%)	25 (81%)	6 (19%)	25 (81%)	21 (68%)	10 (32%)	30 (97%)	1 (3%)
**GA**	12 (22%)	43 (78%)	5 (9%)	50 (91%)	10 (18%)	45 (82%)	37 (67%)	17 (31%)	52 (95%)	3 (5%)
**AA**	4 (36%)	7 (64%)	4 (36%)	7 (64%)	4 (36%)	7 (64%)	9 (82%)	2 (18%)	10 (91%)	1 (9%)
***p*-value**	0.6		**0.05**		0.4		0.6		0.7	

**Table:** (**b**)

Genotype	RF		Anti-CCP		Anti-Ro60		Anti-Ro52		Anti-His		Anti-dsDNA	
	(−)	(+)	(−)	(+)	(−)	(+)	(−)	(+)	(−)	(+)	(−)	(+)
**INF-G rs1861493 A/G**												
**AA**	29 (76%)	9 (24%)	29 (100%)	0	36 (95%)	2 (5%)	31 (82%)	7 (18%)	34 (89%)	4 (11%)	34 (89%)	4 (11%)
**AG**	16 (47%)	18 (53%)	25 (89%)	3 (11%)	29 (85%)	5 (15%)	22 (65%)	12 (35%)	27 (79%)	7 (21%)	30 (88%)	4 (12%)
**GG**	5 (71%)	2 (29%)	3 (100%)	0	8 (100%)	0	6 (75%)	2 (25%)	7 (87%)	1 (13%)	4 (50%)	4 (50%)
***p*-value**	**0.03**		0.2		0.2		0.3		0.5		**0.01**	
**INF-G rs2069705 A/G**												
**AA**	25 (71%)	10 (29%)	26 (100%)	0	36 (100%)	0	31 (86%)	5 (14%)	32 (89%)	4 (11%)	33 (92%)	3 (8%)
**AG**	27 (55%)	22 (45%)	35 (95%)	2 (5%)	40 (82%)	9 (18%)	33 (67%)	16 (33%)	41 (84%)	8 (16%)	43 (88%)	6 (12%)
**GG**	6 (67%)	3 (33%)	9 (90%)	1 (10%)	10 (100%)	0	8 (80%)	2 (20%)	8 (80%)	2 (20%)	6 (60%)	4 (40%)
***p*-value**	0.3		0.1		**0.009**		0.1		0.7		**0.02**	
**IFN-G rs2069718 G/A**												
**GG**	20 (67%)	10 (33%)	21 (100%)	0	31 (100%)	0	26 (84%)	5 (16%)	27 (87%)	4 (13%)	28 (90%)	3 (10%)
**GA**	33 (60%)	22 (40%)	39 (95%)	2 (5%)	46 (84%)	9 (16%)	39 (71%)	16 (29%)	47 (85%)	8 (15%)	50 (91%)	5 (9%)
**AA**	7 (70%)	3 (30%)	5 (83%)	1(17%)	11 (100%)	0	9 (82%)	2 (18%)	9 (82%)	2 (18%)	7 (64%)	4 (36%)
***p*-value**	**0.08**		0.2		**0.02**		0.4		0.9		**0.04**	

(−) negative, n (%) of MCTD patients without autoantibody presence. (+) positive, n (%) of MCTD patients with autoantibody presence.

## Supplementary Materials

The following are available online at https://www.mdpi.com/2077-0383/8/12/2046/s1, Table S1: Scale of MCTD activity (MCTD-AI); Table S2: Scale of MCTD damage (MCTD-DI); Table S3: SNPs information and genotyping results for MCTD patients and control group.

## References

[1] Sharp G.C., Irvin W.S., Tan E.M., Gould R.G., Holman H.R. (1972). Mixed connective tissue disease—An apparently distinct rheumatic disease syndrome associated with a specific antibody to an extractable nuclear antigen (ENA). Am. J. Med..

[2] Minkin W., Rabhan N. (1976). Mixed connective tissue disease. Arch. Dermatol..

[3] Ortega-Hernandez O.D., Shoenfeld Y. (2012). Mixed connective tissue disease: An overview of clinical manifestations, diagnosis and treatment. Best Pract. Res. Clin. Rheumatol..

[4] Aringer M., Steiner G., Smolen J.S. (2005). Does mixed connective tissue disease exist? Yes. Rheum. Dis. Clin. N. Am..

[5] Greidinger E.L., Hoffman R.W. (2005). Autoantibodies in the pathogenesis of mixed connective tissue disease. Rheum. Dis. Clin. N. Am..

[6] Tani C., Carli L., Vagnani S., Talarico R., Baldini C., Mosca M., Bombardieri S. (2014). The diagnosis and classification of mixed connective tissue disease. J. Autoimmun..

[7] Kattah N.H., Kattah M.G., Utz P.J. (2010). The U1-snRNP complex: Structural properties relating to autoimmune pathogenesis in rheumatic diseases. Immunol. Rev..

[8] Casciola-Rosen L.A., Miller D.K., Anhalt G.J., Rosen A. (1994). Specific cleavage of the 70-kDa protein component of the U1 small nuclear ribonucleoprotein is a characteristic biochemical feature of apoptotic cell death. J. Biol. Chem..

[9] Paradowska-Gorycka A. (2015). U1-RNP and TLR receptors in the pathogenesis of mixed connective tissue disease. Part I. The U1-RNP complex and its biological significance in the pathogenesis of mixed connective tissue disease. Reumatologia.

[10] Keith M.P., Moratz C., Tsokos G.C. (2007). Anti-RNP immunity: Implications for tissue injury and the pathogenesis of connective tissue disease. Autoimmun. Rev..

[11] Hof D., Raats J.M., Pruijn G.J. (2005). Apoptotic modifications affect the autoreactivity of the U1 snRNP autoantigen. Autoimmun. Rev..

[12] Hoffman R.W., Maldonado M.E. (2008). Immune pathogenesis of Mixed Connective Tissue Disease: A short analytical review. Clin. Immunol..

[13] Li J., Wang X., Zhang F., Yin H. (2013). Toll-like receptors as therapeutic targets for autoimmune connective tissue diseases. Pharmacol. Ther..

[14] Ronnblom L. (2016). The importance of the type I interferon system in autoimmunity. Clin. Exp. Rheumatol..

[15] Bengtsson A.A., Rönnblom L. (2017). Role of interferons in SLE. Best Pract. Res. Clin. Rheumatol..

[16] Psarras A., Emery P., Vital E.M. (2017). Type I interferon-mediated autoimmune diseases: Pathogenesis, diagnosis and targeted therapy. Rheumatology (Oxford).

[17] Chen J.Y., Wang C.M., Chen T.D., Jan Wu Y.J., Lin J.C., Lu L.Y., Wu J. (2018). Interferon-λ3/4 genetic variants and interferon-λ3 serum levels are biomarkers of lupus nephritis and disease activity in Taiwanese. Arthritis Res. Ther..

[18] McNab F., Mayer-Barber K., Sher A., Wack A., O’Garra A. (2015). Type I interferons in infectious disease. Nat. Rev. Immunol..

[19] Eloranta M.L., Alm G.V., Ronnblom L. (2013). Disease mechanisms in rheumatology–tools and pathways: Plasmacytoid dendritic cells and their role in autoimmune rheumatic diseases. Arthritis Rheum..

[20] Yu S.L., Chan P.K., Wong C.K., Szeto C.C., Ho S.C., So K., Yu M.M., Yim S.F., Cheung T.H., Wong M.C. (2012). Antagonist-mediated down-regulation of Toll-like receptors increases the prevalence of human papillomavirus infection in systemic lupus erythematosus. Arthritis Res. Ther..

[21] Chauhan S.K., Singh V.V., Rai R., Rai M., Rai G. (2013). Distinct autoantibody profiles in systemic lupus erythematosus patients are selectively associated with TLR7 and TLR9 upregulation. J. Clin. Immunol..

[22] Midgley A., Thorbinson C., Beresford M.W. (2012). Expression of Toll-like receptors and their detection of nuclear self-antigen leading to immune activation in JSLE. Rheumatology (Oxford).

[23] El-Sherbiny Y.M., Psarras A., Md Yusof M.Y., Hensor E.M.A., Tooze R., Doody G., Mohamed A.A.A., McGonagle D., Wittmann M., Emery P. (2018). A novel two-score system for interferon status segregates autoimmune diseases and correlates with clinical features. Sci. Rep..

[24] Ramos P.S., Williams A.H., Ziegler J.T., Comeau M.E., Guy R.T., Lessard C.J., Li H., Edberg J.C., Zidovetzki R., Criswell L.A. (2011). Genetic analyses of interferon pathway-related genes reveal multiple new loci associated with systemic lupus erythematosus. Arthritis Rheum..

[25] Kariuki S.N., Ghodke-Puranik Y., Dorschner J.M., Chrabot B.S., Kelly J.A., Tsao B.P., Kimberly R.P., Alarcón-Riquelme M.E., Jacob C.O., Criswell L.A. (2015). Genetic analysis of the pathogenic molecular sub-phenotype interferon-alpha identifies multiple novel loci involved in systemic lupus erythematosus. Genes Immun..

[26] Niewold T.B. (2011). Interferon alpha as a primary pathogenic factor in human lupus. J. Interferon Cytokine Res..

[27] Ghodke-Puranik Y., Dorschner J.M., Vsetecka D.M., Amin S., Makol A., Ernste F., Osborn T., Moder K., Chowdhary V., Eliopoulos E. (2017). Lupus-Associated Functional Polymorphism in PNP Causes Cell Cycle Abnormalities and Interferon Pathway Activation in Human Immune Cells. Arthritis Rheumatol..

[28] Ghodke-Puranik Y., Imgruet M., Dorschner J.M., Shrestha P., McCoy K., Kelly J.A., Marion M., Guthridge J.M., Langefeld C.D., Harley J.B. (2019). Novel genetic associations with interferon in systemic lupus erythematosus identified by replication and fine-mapping of trait-stratified genome-wide screen. Cytokine.

[29] Morris D.L., Sheng Y., Zhang Y., Wang Y.F., Zhu Z., Tombleson P., Chen L., Cunninghame Graham D.S., Bentham J., Roberts A.L. (2016). Genome-wide association meta-analysis in Chinese and European individuals identifies ten new loci associated with systemic lupus erythematosus. Nat. Genet..

[30] Teruel M., Alarcon-Riquelme M.E. (2016). The genetic basis of systemic lupus erythematosus: What are the risk factors and what have we learned. J. Autoimmun..

[31] Järvinen T.M., Hellquist A., Zucchelli M., Koskenmies S., Panelius J., Hasan T., Julkunen H., D′Amato M., Kere J. (2012). Replication of GWAS-identified systemic lupus erythematosus susceptibility genes affirms B-cell receptor pathway signaling and strengthens the role of IRF5 in disease susceptibility in a Northern European population. Rheumatology (Oxford).

[32] Skaug B., Assassi S. (2019). Type I interferon dysregulation in Systemic Sclerosis. Cytokine.

[33] Carnero-Montoro E., Barturen G., Povedano E., Kerick M., Martinez-Bueno M., Ballestar E., Martin J., Teruel M., Alarcón-Riquelme M.E., Precisesads Clinical Consortium (2019). Epigenome-Wide Comparative Study Reveals Key Differences Between Mixed Connective Tissue Disease and Related Systemic Autoimmune Diseases. Front. Immunol..

[34] Carnero-Montoro E., Alarcón-Riquelme M.E. (2018). Epigenome-wide association studies for systemic autoimmune diseases: The road behind and the road ahead. Clin. Immunol..

[35] Teruel M., Sawalha A.H. (2017). Epigenetic variability in systemic lupus erythematosus: What we learned from genome-wide DNA methylation studies. Curr. Rheumatol. Rep..

[36] Hof D., Cheung K., de Rooij D.J., van den Hoogen F.H., Pruijn G.J., van Venrooij W.J., Raats J.M. (2005). Autoantibodies specific for apoptotic U1-70K are superior serological markers for mixed connective tissue disease. Arthritis Res. Ther..

