# Supplementary material for: Interferons (IFN-A/-B/-G) Genetic Variants in Patients with Mixed Connective Tissue Disease (MCTD)
Source: J Clin Med. 2019 Nov 21;8(12):2046. doi: 10.3390/jcm8122046 (PMC6947393; doi:10.3390/jcm8122046)
Supplement: Supplementary file 1 [file jcm-08-02046-s001.pdf]

Table S1. Scale of MCTD activity (MCTD-AI).

Point value	Clinical manifestation
8	Pulmonary involvment
8	Pulmonary hypertension
8	Vasculitis
4	Esophageal involvement
4	Myositis
4	Neuropathy
4	Kidney involvement
2	Skin symptoms
2	Swollen hands
2	Polyarthritis/
2	Serositis
1	↓ platelets
	↓ leucocytes
1	↓ Hb
1	↑ ESR/CRP
1	↑ gamma globulins

Table S2. Scale of MCTD damage (MCTD-DI).

Type of symptom	Clinical manifestation
Eye symptoms (ophthalmoscopic confirmed)	Cataract
	Changes in the retina or optic atrophy
Neuropsychiatric symptoms	Cognitive impairment
	Convulsions requiring treatment ≥6 months
	Stroke
	Neuropathy of the cranial or peripheral nerves (excluding the optic nerve)
	Transverse myelitis
Kidney involvement	GFR <50%
	Proteinuria >3,5 g/24 h
	End-stage renal disease
Lung involvement	Pulmonary hypertension (> 10% from the upper limit of normal)
	Pulmonary fibrosis (confirmed by X-ray / CT / biopsy)
	"Contracted lungs" syndrome
	↓ DLCO
	↓ FEV1
Cardiovascular Symptoms	NT treated> 6 months
	Cardiomyopathy / ventricular dysfunction (documented in echocardiography)
	Pericarditis> 6 months / pericardiectomy
	Coronary artery disease or coronary artery bypass
	Myocardial infarction
	Valve disease
Peripheral vascular involvement	Chromating for at least 6 months
	Minor tissue loss (finger tip defects)
	Significant tissue loss (e.g. loss of finger or limb)
	Venous / arterial thrombosis with edema, venous stasis or ulceration
Gastrointestinal symptoms	Infarction / resection of the stomach / intestine or other abdominal organ (whenever and for any reason)
	Dysfunction - esophageal dysfunction documented by X-ray
	Impaired gastrointestinal motility, constipation, diarrhea or abdominal pain
	Chronic peritonitis
Musculoskeletal symptoms	Muscle atrophy (reduction of muscle mass assessed by clinical examination)
	Muscle weakness not resulting from active muscle disease
	Deforming or erosive arthritis (including removable deformations)
	Osteoporosis with peripheral / compression fractures of the vertebrae

	(excluding aseptic necrosis)
	Non-fracture osteoporosis requiring anti-osteoporotic treatment (except CaCO3 / vitamin D3)
	Aseptic necrosis
Skin symptoms	Alopecia with scarring
	Extensive scarring or skin atrophy (except for scalp and fingertips)
	Skin ulcers (excluding thrombosis) for at least 6 months
	Skin calciferum / subcutaneous tissue / soft tissue
	Fat loss (local / diffuse)
Endocrine Disorders	Growth retardation (children)
	Delayed development of secondary sex characteristics (children)
	Hypertrichosis
	Irregular periods
	Primary or secondary amenorrhea
	Diabetes
	Infertility (adults)
	Sexual dysfunction (adults)
Infections	Chronic infections
	Multiple infections (> 3 infections at the same / different location within 6 months)
Tumor	Cancer (specify type, stage)
Others	Death (cause, date)

Table S3. SNPs information and genotyping results for MCTD patients and control group.

SNP ID	Allele	SNP Type	MAF			p (HWE)	
			MCTD	Control	1000 Genome-EUR	MCTD	Control
**IFN-A rs10757212**	G/A	exon	0.17	0.22	0.19	0.36	0.31
IFN-A rs3758236	T/A	intron	0.11	0.18	0.15	1	0.15
IFN-B rs7873167	T/G	intragenic	0.11	0.11	0.11	0.62	0.8
IFN-B rs10964831	A/T	5′-UTR	0.16	0.15	0.17	0.33	0.36
INF-G rs1861493	A/G	intron	0.31	0.27	0.27	1	0.81
IFN-G rs2069705	A/G	5′-UTR	0.36	0.45	0.33	0.44	0.91
IFN-G rs2069718	G/A	intron	0.40	0.52	0.40	0.02	0.29

MAF - minor allele frequency; HWE - Hardy-Weinberg equilibrium; EUR - European; UTR – untranslated region.
